# Interlayer Modification of Pseudocapacitive Vanadium Oxide and Zn(H_2_O)_n_^2+^ Migration Regulation for Ultrahigh Rate and Durable Aqueous Zinc‐Ion Batteries

**DOI:** 10.1002/advs.202004924

**Published:** 2021-05-24

**Authors:** Hangda Chen, Juanjuan Huang, Shuhao Tian, Li Liu, Tianfeng Qin, Lei Song, Yanpeng Liu, Yanan Zhang, Xiaogang Wu, Shulai Lei, Shanglong Peng

**Affiliations:** ^1^ National & Local Joint Engineering Laboratory for Optical Conversion Materials and Technology School of Physical Science and Technology Lanzhou University Lanzhou 730000 P.R. China; ^2^ Department of Applied Physics Eindhoven University of Technology Eindhoven 5600 MB the Netherlands; ^3^ Hubei Key Laboratory of Low Dimensional Optoelectronic Materials and Devices Hubei University of Arts and Science Xiangyang Hubei 441053 China

**Keywords:** aqueous zinc‐ion batteries, high‐rate performance, hydrated vanadium oxide, hydrated zinc‐ion, pseudocapacitance

## Abstract

The interlayer modification and the intercalation pseudocapacitance have been combined in vanadium oxide electrode for aqueous zinc‐ion batteries. Intercalation pseudocapacitive hydrated vanadium oxide Mn_1.4_V_10_O_24_·12H_2_O with defective crystal structure, interlayer water, and large interlayer distance has been prepared by a spontaneous chemical synthesis method. The inserted Mn^2+^ forms coordination bonds with the oxygen of the host material and strengthens the interaction between the layers, preventing damage to the structure. Combined with the experimental data and DFT calculation, it is found that Mn^2+^ refines the structure stability, adjusts the electronic structure, and improves the conductivity of hydrated vanadium oxide. Also, Mn^2+^ changes the migration path of Zn^2+^, reduces the migration barrier, and improves the rate performance. Therefore, Mn^2+^‐inserted hydrated vanadium oxide electrode delivers a high specific capacity of 456 mAh g^−1^ at 0.2 A g^–1^, 173 mAh g^–1^ at 40 A g^–1^, and a capacity retention of 80% over 5000 cycles at 10 A g^–1^. Furthermore, based on the calculated zinc ion mobility coefficient and Zn(H_2_O)_n_^2+^ diffusion energy barrier, the possible migration behavior of Zn(H_2_O)_n_^2+^ in vanadium oxide electrode has also been speculated, which will provide a new reference for understanding the migration behavior of hydrated zinc‐ion.

## Graphical abstract

The strategy of interlayer modification of Mn^2+^ is applied to regulate the interlayer atomic coordination structure, electronic structure, and affect Zn^2+^ migration behavior in the vanadium oxide (V_10_O_24_·nH_2_O). Zinc ion intercalation pseudocapacitance, ultra‐high rate performance, and cycling stability are achieved and the Zn(H_2_O)_n_^2+^ migration behavior is analyzed and speculated.

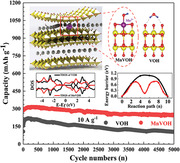

## 1 Introduction

Lithium‐ion batteries have been successfully commercialized due to their high working voltage and high energy density.^[^ ^1^ ^]^ However, the current development of lithium‐ion batteries is limited by the lithium scarcity, insecurity, and price of lithium ore. Therefore, it is essential to develop a new type of ion batteries to replace the lithium‐ion batteries. Multivalent ions such as Mg^2+^, Zn^2+^, Ca^2+^, and Al^3+^ can carry more charges per ion than that of the Li^+^, and they are believed to be promising working ions in rechargeable batteries.^[^ ^2^, ^3^, ^4^, ^5^ ^]^ Among all multivalent ions mentioned above, zinc metals as anode electrode material for zinc‐ion batteries (ZIBs), have a low redox potential (−0.76 vs standard hydrogen electrode), high capacity (820 mAh g^−1^), easy production, high abundance, and intrinsic safety. So aqueous ZIBs have attracted tremendous attention.^[^ ^6^, ^7^, ^8^ ^]^ Recently, a series of cathode materials, such as manganese oxide, vanadium based composites, Prussian blue analogs, metal sulfides, and organic compounds, have been researched for ZIBs.^[^ ^6^, ^7^, ^8^, ^9^, ^10^, ^11^, ^12^, ^13^ ^]^ Among them, vanadium oxide with tunnel, layered or open framework structure, possesses sufficient active site for Zn^2+^ and has been considered as ideal cathode materials for ZIBs. However, the poor conductivity and fragile open framework structure hinder the ion diffusion kinetics and lead to fast performance degradation during cycling.^[^ ^13^, ^14^, ^15^ ^]^ In order to solve the above problems, many strategies have been developed to optimize vanadium‐based cathode materials including pre‐inserted cations/molecules pillaring, structural modification, nanostructuring, and more.^[^ ^10^, ^16^, ^17^ ^]^ For instance, Li et al. synthesized Ni_0.25_V_2_O_5_·nH_2_O by hydrothermal method, which demonstrated a high reversible specific capacity of 402–147 mAh g^–1^ at 0.2‐5 A g^–1^.^[^ ^18^ ^]^ Geng et al. synthesized Mn_0.15_V_2_O_5_·nH_2_O by introducing Mn^2+^ that exhibited high rate performance of 150 mAh g^–1^ at 10 A g^–1^.^[^ ^19^ ^]^ Na_0.33_V_2_O_5_, NaCa_0.6_V_6_O_16_·3H_2_O, V_10_O_24_‐Aland more. have also been introduced by ion pillaring and deliver effectively improved stability of vanadium oxide cathode.^[^ ^20^, ^21^, ^22^ ^]^ However, in these battery‐type intercalation materials, kinetics limited by ion diffusion process and their energy storage performance is still not satisfactory. Also, a type of intercalation pseudocapacitive energy storage is easier to obtain high rate performance, with Faradaic charge transfer without crystallographic phase change.^[^ ^23^ ^]^ Over the past decade, the focus on intercalation pseudocapacitive materials is mainly in nanostructured, high defective, and metastable materials. But the structural stability of these intercalation pseudocapacitive materials is still unsatisfactory.^[^ ^15^, ^24^ ^]^ Therefore, the interlayer tuning strategy and intercalation pseudocapacitive property have been applied to the hydrated vanadium oxide electrode materials, demonstrating an effective way to obtain high stability and rate performance.

## 2 Results and Discussion

**Figure 1:**
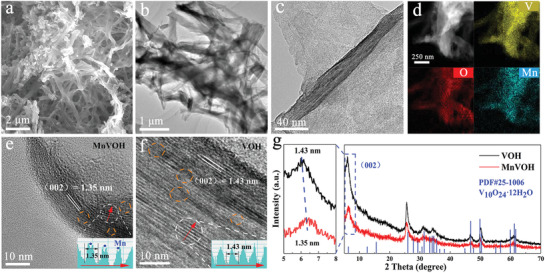
a–c) SEM and TEM of MnVOH. d) EDS mapping of MnVOH. e,f) HRTEM of MnVOH and VOH; the yellow circle represents lattice defects. g) XRD patterns of MnVOH and VOH.

Mn_1.4_V_10_O_24_·nH_2_O (MnVOH) was synthesized by a mild spontaneous chemical reaction, and it exhibits a cobweb‐like crisscross microscopic morphology as shown in **Figure** **1**a–c, which is conducive to prevent self‐aggregation and electrolyte penetration. V_10_O_24_·nH_2_O (VOH) also exhibits a similar morphology with MnVOH (Figure S1, Supporting Information) and the specific surface area of them is close with a value of 5.4129 m² g^−1^ for MnVOH and 3.4398 m² g^−1^ for VOH. As shown in Figure 1d, energy dispersive spectrometer mapping indicates that Mn^2+^ is uniformly distributed in MnVOH and the mass ratio of V to Mn is about 7:1 (Figure S2, Supporting Information). High‐resolution‐transmission electron microscopy (HRTEM) identifies the interlayer distance of 13.5 Å for MnVOH and 14.3 Å for VOH, symbolizing the (002) plane of nanosheets (Figure 1e,f). The insets of Figure 1e,f are the image intensity along the red arrow. VOH presents periodic single‐peak change and MnVOH presents periodic bimodal change, and it can be inferred that Mn^2+^ may exist between layers.^[^ ^25^ ^]^ Some defects are observed as shown in yellow circle (Figure 1e,f), which is conducive to intercalation pseudocapacitive behavior.^[^ ^26^, ^27^ ^]^ From the X‐ray diffraction (XRD) pattern in Figure 1g, all the diffraction peaks of VOH are well indexed to the phase of V_10_O_24_·nH_2_O (PDF#25‐1006) and MnVOH is determined to be Mn_1.4_V_10_O_24_·nH_2_O. According to the Bragg's equation, the shift of the (002) peak from 6.1 to 6.3 degrees means that the interlayer distance is reduced from 14.3 Å to 13.5 Å, which is consistent with the HRTEM results.

**Figure 2:**
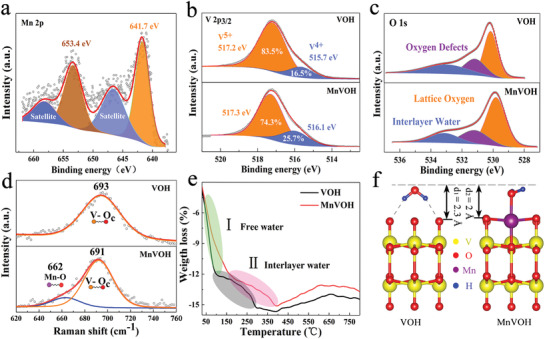
a) XPS spectrum of Mn 2p. b,c) The XPS spectrum of V 2p_3/2_ and O 1s of both samples. d) The partially fitted Raman spectra; the peak at 662 cm^–1^ demonstrates a Mn—O coordination bonds between Mn^2+^ and O^2–^. e) Thermogravimetric Analysis curves of VOH and MnVOH. f) Optimized geometries of H_2_O adsorbed on VOH and MnVOH surface.

X‐ray photoelectron spectroscopy (XPS) was further used to analyze chemical valences. The peaks of Mn 2p spectrum in **Figure** **2**a show that Mn ions are divalent,^[^ ^28^ ^]^ which leads to more V^4+^ content in MnVOH as shown in Figure 2b. The peaks of O 1s spectrum in Figure 2c represent interlayer water, oxygen defects, and lattice oxygen, respectively. Some oxygen defects in both samples can facilitate the adsorption of Zn^2+^ and promote the intercalation pseudocapacitive behavior.^[^ ^24^, ^25^, ^26^ ^]^ The Fourier Transform infrared spectroscopy and Raman spectra (Figure S3, Supporting Information) were used to characterize the bonding information. In Raman spectra of Figure 2d, it is worth noting that, there is a weak peak at 662 cm^–1^ corresponding to the vibration of Mn—O bonds for MnVOH based on curve‐fitting with lorentz functions.^[^ ^29^ ^]^ To confirm the effect of Mn^2+^ on the thermal stability, thermogravimetric analysis (TGA) is used to analyze the water loss in the heating process. Both samples were dehydrated in two stages as shown in Figure 2e. The first stage is the loss of free water, and the second stage is the loss of interlayer water.^[^ ^16^ ^]^ MnVOH has a higher temperature of losing interlayer water than that of the VOH, which may be due to the electrostatic interaction between Mn^2+^ and interlayer water, reflecting the better thermal stability of interlayer water for MnVOH.^[^ ^29^ ^]^

According to the previous analysis, VOH has many surface oxygen dangling bonds and is usually not stable enough. Therefore, VOH is easy to adsorb Mn^2+^ to form coordination bonds and water to form hydrogen bonds.^[^ ^30^, ^31^ ^]^ Our DFT calculation demonstrates that the inserted Mn^2+^ not only saturates [VO] surface dangling bonds to improve its stability, but also reduces interlayer distance (Figure 2f) and strengthens the interlayer interaction by forming strong coordination bonds, which agrees well with our experimental observations mentioned above.

**Figure 3:**
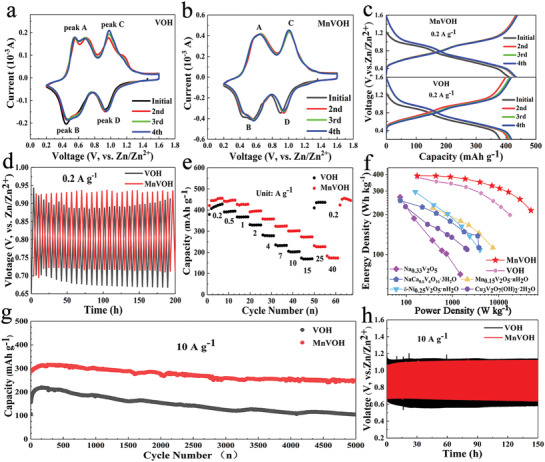
a,b) Initial three CV curves of VOH and MnVOH collected at a sweep rate of 0.1 mV s^–1^. c) Initial three charge–discharge curves of VOH and MnVOH at 0.2 A g^–1^. d) Average dis‐/charge voltage at 0.2 A g^–1^ of both samples. e) Rate capability at various current densities. f) Ragone plot of the Zn‐ion batteries with different cation ion pre‐inserted vanadium oxide. g) Cycling stability tested at 10 A g^–1^. h) Average dis‐/charge voltage at 10 A g^–1^.

In order to analyze the electrochemical reaction of cathode electrode, cyclic voltammetry (CV) measurements at a voltage range of 0.2–1.6 V (0.1 mV s^–1^) were obtained with typical coin‐type cell. Two pairs of redox peaks in both samples are displayed in **Figure** **3**a,b. The reaction of the V^4+^/V^3+^ appears around 0.6 V, and the peak pair at higher voltage around 1 V is related to the redox of V^5+^/V^4+^.^[^ ^14^, ^32^, ^33^ ^]^ It is worth noting that the broad peaks on CV curves and pseudo linear voltage responses on galvanostatic charge and discharge (GCD) curves (Figure 3c) mean the intercalation pseudocapacitive behavior of Zn^2+^. Manganese ion remains divalent during charging and discharging, indicating that Mn^2+^ does not participate in the reaction (Figure S4, Supporting Information). The ex situ XRD of MnVOH (Figure S5, Supporting Information) shows that there is no phase change and the morphology remains unchanged during charging and discharging process (Figure S6, Supporting Information). And the MnVOH cathode also delivers the intercalation pseudocapacitive behavior of Zn^2+^.^[^ ^15^, ^34^ ^]^ Although water‐based electrolytes may all have the influence of H^+^, from the ex situ XRD (Figure S5, Supporting Information) and SEM at different vlotage (Figure S6, Supporting Information), no obvious by‐products are found, so it is speculated that H^+^ is not involved in the reaction. The reversible movement of the (002) peak during charging and discharging demonstrates the reversibility of Zn^2+^ intercalation/de‐intercalation between the interlayer of MnVOH. The XPS spectrum of Zn 2p for MnVOH at 0.2 V/1.6 V also proves the reversible migration of zinc ions (Figure S7, Supporting Information). In addition, the initial three cycles of CV and GCD curves of MnVOH are more stable than that of VOH, which may be attributed to that Mn—O bonds of MnVOH which improve lattice stability. The unstable average charge–discharge voltage of VOH at 0.2 A g^−1^ in Figure 3d also reflects that the zinc ions damage the crystal structure when deeply de/intercalacion.

In order to evaluate the electrochemical energy storage performance of the batteries, the rate and high current density charging–discharging were conducted. For the rate capability tests as shown in Figure 3e, the VOH electrode delivers a discharge specific capacity of 397 mAh g^–1^ for the second cycle and reached 427 mAh g^–1^ for 7th cycle at 0.2 A g^–1^. More notably, MnVOH electrode demonstrates excellent rate performance. With the current density increasing from 0.2 to 40 A g^–1^ and then decreasing back to 0.2 A g^–1^, MnVOH electrode delivers specific capacities of 456, 447, 428, 396, 358, 324, 302, 273, 227, 173, and 456 mAh g^–1^, respectively. And VOH electrode exhibits capacities of 407, 394, 368, 329, 278, 233, 204, 168, and 436 mAh g^–1^ (0.2 to 15 and 0.2 A g^–1^). Compared with the capacities at 0.2 A g^–1^, the capacity retention of MnVOH electrode is 60% at 15 A g^–1^, which is higher than that of VOH electrode (42%). The MnVOH electrode delivers a similar specific capacity at small current density of 0.2 A g^−1^ (Figure S8, Supporting Information) and a higher specific capacity at high current density of 2 A g^–1^ (Figure S9, Supporting Information) than that of VOH electrode. This shows that Mn^2+^ mainly promotes the rapid migration ability of Zn^2+^ instead of providing more Zn^2+^ adsorption and storage sites. Correspondingly, MnVOH electrode exhibits a high energy‐density/power‐density of 391 Wh kg^−1^/ 172 W kg^−1^ and 214 Wh kg^−1^/ 49.6 kW kg^−1^ at 0.2 A g^−1^ and 40 A g^−1^ (based on the mass of MnVOH) as depicted in Figure 3f, which is much better than that of many recently reported ZIBs cathodes such as Na_0.33_V_2_O_5_,^[^ ^35^ ^]^ NaCa_0.6_V_6_O_16_·3H_2_O,^[^ ^20^ ^]^ Mn_0.15_V_2_O_5_·nH_2_O,^[^ ^19^ ^]^ *δ*‐Ni_0.25_V_2_O_5_·nH_2_O,^[^ ^18^ ^]^ and Cu_3_V_2_O_7_(OH)_2_·2H_2_O.^[^ ^36^ ^]^ And the stability of the MnVOH electrode (10 A g^–1^) is shown in Figure 3g and 80% capacity is retained after 5000 cycles. The initial increase in capacity may be the activation process of the electrode, since the reaction mechanism has not changed because of the constant‐shape of GCD and CV curves in initial cycles (Figure S10, Supporting Information). The excellent rate and stability performance of MnVOH exceeds that of similar materials previously reported.^[^ ^36^, ^37^ ^]^ Since both electrode materials of VOH and MnVOH have similar characteristics, such as large interlayer spacing and oxygen defects, the difference in rate performance may be due to the insertion of Mn^2+^. The average charge–discharge voltage during the cycles at 10 A g^–1^ are displayed in Figure 3h, the more stable voltage of MnVOH may be due to the fact that the Mn—O bond strengthens the interaction between layers and makes the layers more stable when a large amount of Zn^2+^ migrates rapidly.^[^ ^38^ ^]^ In addition, the closer average charge–discharge voltage indicates that MnVOH has a smaller polarization and better reaction kinetics in the electrochemical processes, which is consistent with the high rate performance of MnVOH. ^[^ ^29^, ^39^ ^]^

**Figure 4:**
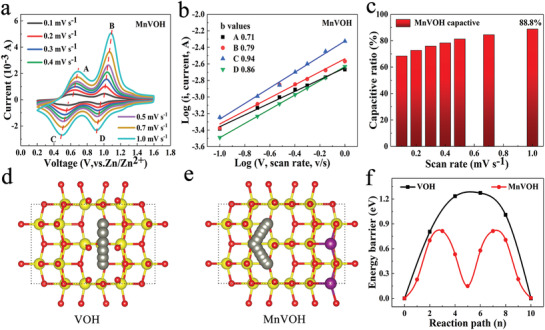
a) CV curves of MnVOH at multiple scan rates of MnVOH. b) Log (*i*) versus log (*v*) plots of four redox peaks in CV curves of MnVOH. c) The percentages of capacitive contributions at different scan rates. Schematic diagram of the diffusion path of zinc ions between layers, d) VOH and e) MnVOH. f) The corresponding diffusion energy barrier of (d) and (e).

In order to explain the electrochemical kinetics of the MnVOH cathode, the CV curve was tested at different scanning speeds of 0.1~1 mV s^−1^. As the electrode polarization becomes more serious at the large‐scan speed, a voltage shift occurred in cathodic/anodic peaks as shown in **Figure** **4**a. It is generally considered that the current consisting of diffusion and capacitive, refers to the following formula:

(1) $$i=av^{b}$$

In the above equation, *v* refers to the scan rate (mV s^−1^), *a* and *b* are adjustable parameters. When *b* is close to 1, it reveals that this process is controlled by the capacitive process. When *b* is close to 0.5, it reveals that this process is controlled by the diffusion process. The *b* values of MnVOH are 0.71, 0.79, 0.94, and 0.86 respectively (Figure 4b), indicating that it is less restricted by diffusion‐controlled process.^[^ ^14^ ^]^ The corresponding proportions of different types of capacity follow the following formula:

(2) $$i=k_{1}v+k_{2}v^{1/2}$$

where *k*_1_ and *k*_2_ are the coefficients of capacitive and diffusion contributions. With the increase of scanning speed, the proportion of capacitance increases and reaches 88.8% at 1 mV s^−1^ (Figure 4c). High proportion of capacitance and *b* values both show that MnVOH has the property of intercalation pseudocapacitance.^[^ ^27^ ^]^

It is universally affirmed that the kinetic limiting step of insertion‐type materials is the solid‐state diffusion. Therefore, the influence of Mn^2+^ on the migration path of Zn^2+^ has been theoretically analyzed. The results show that because the adsorption force of Mn^2+^ on the [VO] surface is stronger than that of Zn^2+^ (Supporting Information for details), the migration path of Zn^2+^ is changed as shown in Figure 4d,e. The inserted Mn^2+^ may cause Zn^2+^ migration path to change and it also occupies the adsorption sites of Zn^2+^ to a certain extent. Therefore, Mn^2+^ only improves the rate performance, but does not significantly increase the capacity. Further calculations show that the energy barrier corresponding to the migration path of Zn^2+^ in MnVOH is lower than that of VOH as placed in Figure 4f. The relationship between Zn^2+^ diffusion coefficient (*D*_Zn_) and the migration energy barrier can be explained by the following formula:^[^ ^40^ ^]^

(3) $$D=p\lambda^{2}v^{*}\exp\left(-E_{B}/\mathrm{kT}\right)$$

where *p* is a geometrical factor, v^*^ is the vibrational frequency, *k* is the hopping distance, and *E*_B_ is the migration energy barrier.^[^ ^40^ ^]^ Therefore, the low energy barrier can make the diffusion of Zn^2+^ in MnVOH easier.

**Figure 5:**
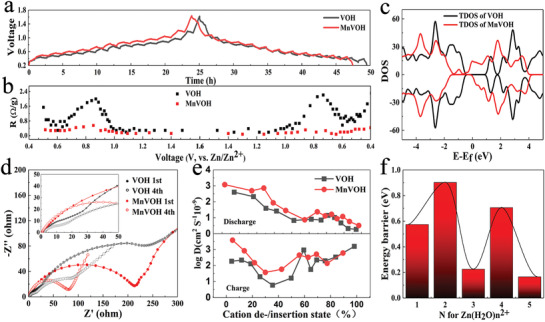
a) GITT plot collected at a current density of 0.2 A g^−1^. b) The reaction resistance during the charging and discharging process. c) Calculated total density of states (DOS) of VOH and MnVOH. d) EIS spectra of VOH and MnVOH before and after the initial four cycles at 0.2 A g^−1^. e) Zn^2+^ diffusion coefficient of both samples during the charging and discharging process calculated by GITT of (a). f) Transition energy barrier of Zn(H_2_O)_n_^2+^ in VOH with the migration path as described in Figure 4d.

To further confirm the previous simulation results, the galvanostatic intermittence titration technique (GITT) was executed. The GITT plots (**Figure** **5**a) were collected at a current density of 0.2 A g^–1^, at which the capacity of them are similar. The reaction resistance (RR) is calculated using the internal resistance (IR) drop.^[^ ^29^ ^]^ The RR of VOH fluctuates regularly with the maximum RR value of about 2.3 Ω g^–1^ which is placed in Figure 5b. The same trend also occurs for MnVOH, but MnVOH has lower and smoother RR values around 0.3 Ω g^–1^. The better conductivity of MnVOH may be caused by the Mn^2+^ insertion, which is well confirmed by subsequent simulation results as depicted in Figure 5c. The calculation result shows that VOH is a semiconductor and has the band gap of 1.76 eV, which is not conducive to batteries performance. Due to the insertion of Mn^2+^ to VOH substrate, highest occupied molecular orbital (LUMO) orbitals of VOH are occupied and MnVOH displays good electronic conductivity.

At the same time, electrochemical impedance spectroscopy (EIS) as depicted in Figure 5d shows that MnVOH has a smaller charge transfer resistance (*R*_ct_),^[^ ^41^ ^]^ which confirmed the low RR of MnVOH. The *R*_CT_ may be related to the activation energy (*E*_a_) for charge transfer during intercalation:^[^ ^40^ ^]^

(4) $$R_{\mathrm{CT}}=A_{o}\exp\left(E_{a}/kT\right)$$

Here, *A*_0_ is a pre‐exponential constant, *k* is the ideal gas constant, and *T* is the temperature. The activation energy of charge transfer is related to the energy required for cationic solvent removal.^[^ ^40^ ^]^ Therefore, the low *R*_CT_ of MnVOH represents a low activation energy, indicating that the energy required to desolvate Zn(H_2_O)_6_^2+^ in MnVOH is lower, which promotes the insertion of Zn^2+^ into MnVOH. Therefore, the adjustment of the electronic structure of VOH by Mn^2+^ improves the electronic conductivity and further promotes the rate performance.

The GITT also was used to calculate the zinc‐ion diffusion coefficients (*D*_Zn_), as shown in Figure 5e. MnVOH electrode shows a higher *D*_Zn_ than that of VOH, which is consistent with its better rate performance. Structural simulation calculations are applied to explore the factors that may lead to changes of *D*_Zn_. The simulation result indicates that hydration number can strongly affect the diffusion energy barrier *E*_B_ of Zn(H_2_O)_n_^2+^, as shown in Figure 5f. Due to the bonding interactions between d orbitals of Zn^2+^ and ligand orbitals of oxygen atoms of H_2_O and VOH, as shown in Figure S11 (Supporting Information) which forms tetrahedral coordination configuration with *n* = 3 and octahedral coordination configurations with *n* = 5. Zn(H_2_O)_n_^2+^ has lower ligand field stabilization energy than other cases (*n* = 1, 2, and 4), as described in crystal field theory. According to the formula (3), it can be inferred that the bound water can promote the migration of Zn^2+^ by reducing the energy barrier. More detailed discussion of the relationship between Zn(H_2_O)_n_^2+^ and *D*_Zn_ will be demonstrated in another work since more calculations and experimental proof should be supply.

## 3 Conclusions

In this work, the strategy of interlayer modification by inserting Mn^2+^ into intercalation pseudocapacitive vanadium oxide has been adopted to refine the electrochemical environment and improve Zn^2+^ storage performance of zinc‐ion batteries. As the cathode, Mn^2+^‐inserted hydrated vanadium oxide demonstrates Zn^2+^ intercalation pseudocapacitive mechanism and delivers an excellent rate performance (40 A g^−1^, 173 mAh g^−1^) and cycle life (80%, 5000 cycles). The effects of Mn^2+^ insertion on the structure, Zn^2+^ migration behavior, and Zn^2+^ storage mechanism have been analyzed. First, Mn^2+^ forms a coordination bond with the oxygen of [VO] layers, which regulates the electronic structure of VOH and improves conductivity. Second, the strong coordination bonds reduce the interlayer distance and Mn^2+^ as “pillar” stabilizes the host structure. Third, the inserted Mn^2+^ affects the diffusion path of zinc ions and reduces the diffusion barrier. In addition, based on the relationship between the zinc ion diffusion coefficient and the diffusion energy barrier of Zn(H_2_O)_n_^2+^ with different bound water number, it is speculated that the bound water can promote the migration of Zn^2+^ by reducing the energy barrier. This work may provide a new understanding of the migration of zinc ions for ZIBs.

## 4 Experimental Section/Methods

Experimental Section details are in the Supporting Information.

## Conflict of Interest

The authors declare no conflict of interest.

## Supporting information

Supporting Information

## Data Availability

Research data are not shared.
