# Supplementary material for: Interlayer Modification of Pseudocapacitive Vanadium Oxide and Zn(H2O)n 2+ Migration Regulation for Ultrahigh Rate and Durable Aqueous Zinc‐Ion Batteries
Source: Adv Sci (Weinh). 2021 May 24;8(14):2004924. doi: 10.1002/advs.202004924 (PMC8292880; doi:10.1002/advs.202004924)
Supplement: Supplementary file 1 — Supporting Information [file ADVS-8-2004924-s001.pdf]

WILEY-VCH

© 2021 Wiley-VCH GmbH

ADVANCED SCIENCE
Open Access

Supporting Information

for *Adv. Sci.*, DOI: 10.1002/advs.202004924

Interlayer Modification of Pseudocapacitive Vanadium Oxide and $Zn(H_2O)n^{2+}$ Migration Regulation for Ultrahigh Rate and Durable Aqueous Batteries

Hangda Chen[a1], Juanjuan Huang[a1], Shuhao Tian[a], Li Liu[a], Tianfeng Qin[a], Lei Song[b], Yanpeng Liu[a], Yanan Zhang[a], Xiaogang Wu[a], Shulai Lei[c], Shanglong Peng[a*]*

Supporting Information

Interlayer Modification of Pseudocapacitive Vanadium Oxide and $Zn(H_2O)n^{2+}$ Migration Regulation for Ultrahigh Rate and Durable Aqueous Batteries

Hangda Chen[a1], Juanjuan Huang[a1], Shuhao Tian[a], Li Liu[a], Tianfeng Qin[a], Lei Song[b], Yanpeng Liu[a], Yanan Zhang[a], Xiaogang Wu[a], Shulai Lei[c], Shanglong Peng[a*]*

Hangda Chen[a1] and Juanjuan Huang[a1] contribute to the work equally.

Preparation of materials:

$Mn_{1.4}V_{10}O_{24}\cdot 12H_2O$ (MnVOH): All chemicals were used as received without purification. 2.1 g of V_2O_5 (99.5%, Aladdin) was dissolved into 400 mL of DI H_2O with 50 mL of H_2O_2 (30%, Aladdin), and 1 g of $MnSO_4\cdot H_2O$ (98%, Aladdin) was dissolved separately into 40 mL of DI water. The two solutions were admixed and transferred to a 1000 mL beaker and waited 15 days at room temperature. Dark green precipitates were collected by centrifugation and washed with water three times. The collected precipitates were dried at 60 ℃ overnight in a vacuum oven.

$V_{10}O_{24}\cdot 12H_2O$ (VOH): VOH was synthesized with the same procedure and processing conditions without Mn sources and the resulting product was black powder.

Chemical and physical characterization:

The chemical component was analyzed on a multifunctional X-ray photoelectron spectroscope (PHI-5702, Mg Kα X-ray, 1253.6 eV) at an accelerating voltage of 40 kV and a

working current of 40 mA. A Fourier transform infrared spectrometer (FTIR, Nicolet, NEXUS 670) was used to collect the functional groups' stretching or bending information from 400 to 4000 cm^{-1} and Raman spectra were recorded on a micro-Raman spectroscope (JY-HR800, y532-nm wavelength YAG laser). A differential scanning calorimeter (TG-DSC, Linseis, STA Pt1600) was adopted to analyze the thermal stability of the sample within 25-800 ℃ in nitrogen gas. The microstructures of the samples were observed using a scanning electron microscope (SEM, Apero S) at a working voltage of 30 kV and a scanning transmission electron microscope (S/TEM, Tecnai F30) with an accelerating voltage of 300 kV. The surface chemical states of the samples were determined using a Kratos Axis Ultra DLD X-ray Photoelectron Spectroscopy system (XPS) with an AlKa radiation source that was operated at 10 mA and 15 kV, and with a charge neutralizer. The angle between the specimen normal and the spectrometer was 0°. The instrument used for BET test is ASAP 2020M & TriStar 3020 (Micromeritics, Analysis Adsorptive : N_2).

Electrochemical characterization:

For the electrode preparation, the active material was mixed with super-p and polyvinylidene fluoride (PVDF) binder in a weight ratio of 7:2:1 in N-methyl-2-pyrrolidone (NMP) solvent to obtain slurry that was pasted on a current collector -carbon fiber cloth. The prepared electrodes were dried in a vacuum oven at 60 ℃ 12h. The mass loading of active materials is 3-4 mg cm^{-2}. Zn metal was used as the anode and 3 M zinc trifluoromethanesulfonate (98%, $Zn(CF_3SO_3)_2$) aqueous solution was injected into the coin-type cells (CR2032) as the electrolyte. A glass fiber filter (Whatman, Grade GF/D) was used as the separator. Cyclic voltammetry curves (CV) and electrochemical impedance spectroscopy (EIS, 100 kHz to 0.01 Hz, 10 mV amplitude) are examined using an electrochemical workstation (CHI 760E, Chenhua). The galvanostatic intermittent titration technique (GITT) was applied to analyze

the reaction and diffusion kinetics at a current density of 0.2 A g^{-1} and a charge/discharge time is 6 mins, interval of 180 min for each step. The galvanostatic cycling studies are performed using LAND battery testing system (2001A) at room temperature. The working voltage of the cells was set from 0.2 to 1.6 V versus Zn/Zn^{2+}.

Computational details

The frozen-core projector augmented wave approach as implemented in the Vienna *Ab initio* Simulation Package (VASP) was carried out in our first-principles calculations.[1] The generalized gradient approximation (GGA) proposed by Perdew, Burke, and Ernzerhof (PBE) was used for the exchange-correlation effect.[2] The cutoff energy for the plane wave basis was set to 400 eV. The Brillouin zone was sampled with a 2×3×1 k-points mesh according to Monkhorst-Pack scheme.[3] The convergence criteria for the total energy and the maximum force on each atom are less than 10^{-4} eV and 0.02 eV/Å, respectively. In addition, the climbing image nudged elastic band (CI-NEB) method was used to determine the minimum energy diffusion pathways of Zn^{2+} and the corresponding energy barriers.[4]

Statistical analysis

XPS data processing used xpspeak41 software, and the C 1s calibration is 284.6 eV. The data (fig 1g; fig 2a-e; fig 3a-c, f; fig 5) are plotted and fitted with the origin software. The data recording interval of the GCD curve is 0.1 s. For the 10 A g^{-1} high-current long-cycle data (figure 3g), individual abnormal data points have been deleted (may be caused by abnormal shutdown of the test software), the deleted data accounts for no more than 2‰ (10/5000) of the total data. The data (XPS, XRD, Raman, TEM, SEM, EDS, etc.) in the manuscript have been repeated more than 3 times, and the battery capacity test has been repeated at least 6 times.

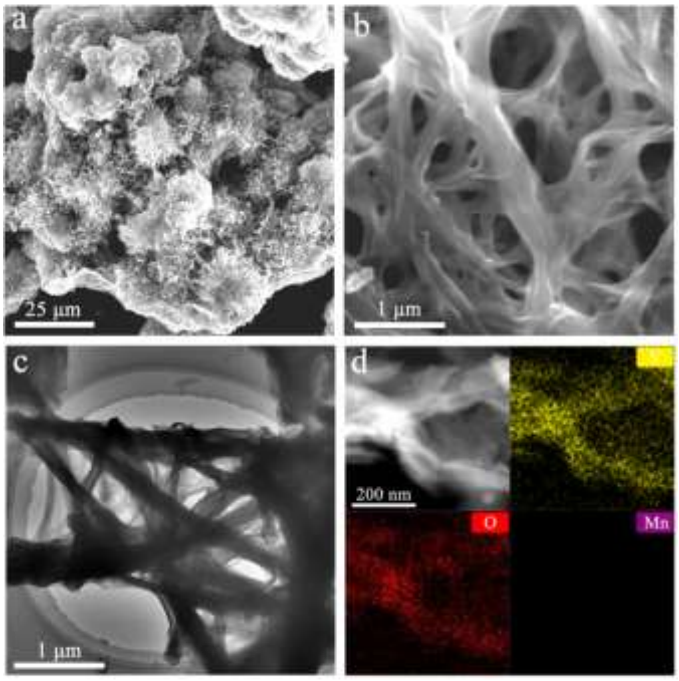

Figure S1 a-c) SEM and TEM of VOH; d) EDS mapping of VOH.

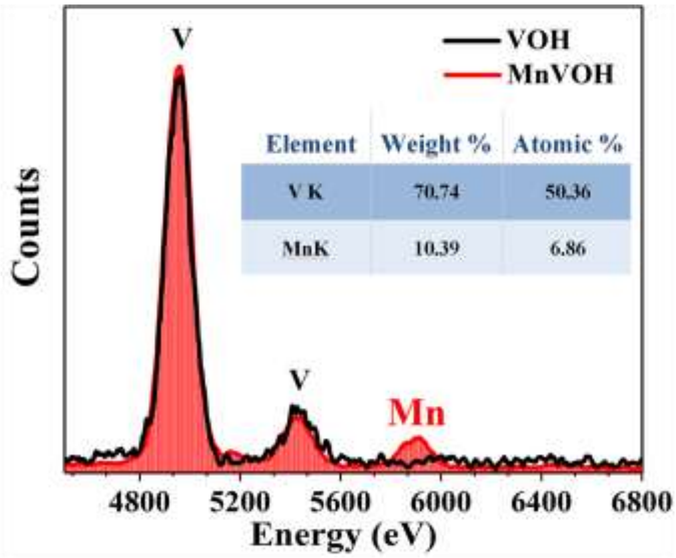

Figure S2 EDS diagram and corresponding element content.

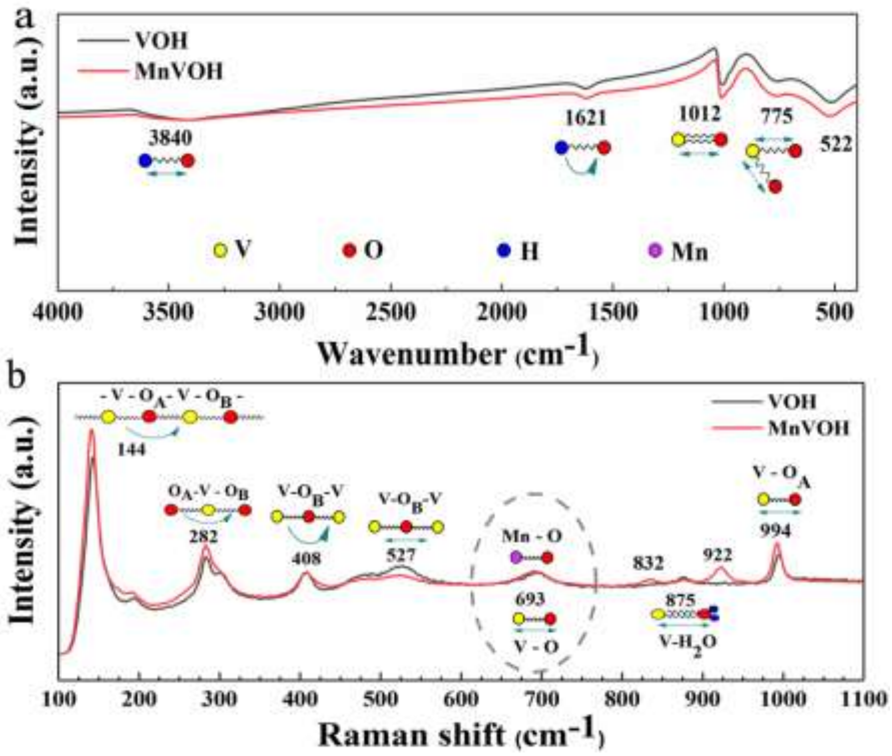

Figure S3 Fourier Transform infrared spectroscopy (a) and Raman spectra; (b) of VOH and MnVOH.

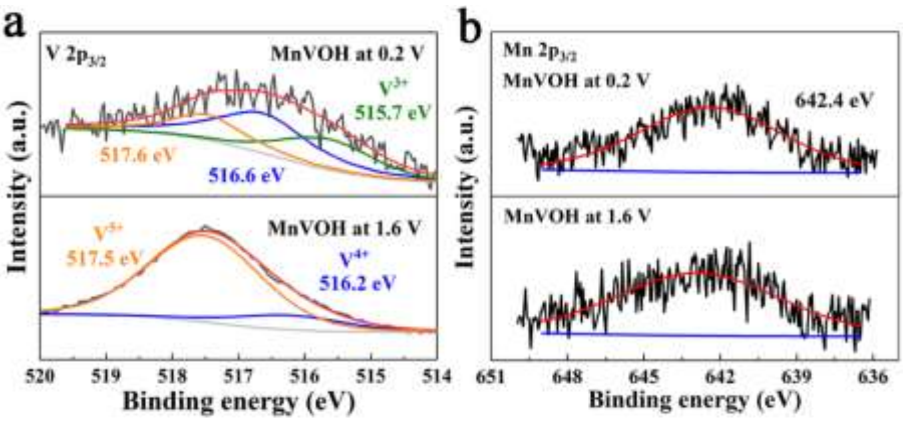

Figure S4 The test sample is MnVOH electrode at 0.2 V or 1.6 V. a) XPS spectrum of V $2p_{3/2}$ at 0.2 V and 1.6V. b) XPS spectrum of Mn $2p_{3/2}$ at 0.2 V and 1.6V. The results show that the binding energy (642.4 eV) of Mn $2p_{3/2}$ remains constant and Mn is both divalent at 0.2 V and 1.6 V, which means Mn^{2+} does not participate in the reaction.

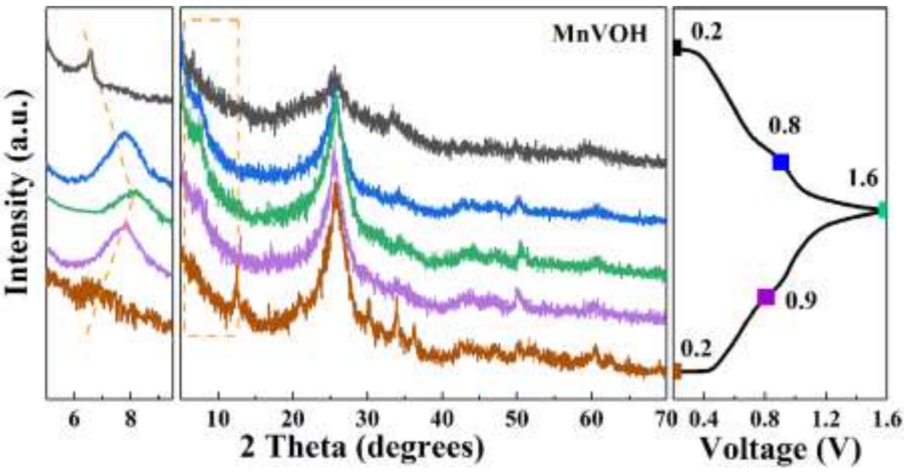

Figure S5 Ex situ XRD patterns of MnVOH during the charged and discharged process.

The data in the small angle (5° ~10°) range have been carefully tested at 0.5° min^{-1}. The shifts and recovery of the characteristic peaks suggest a reversible intercalation reaction occurring in the electrochemical processes with no phase transition and by-products.

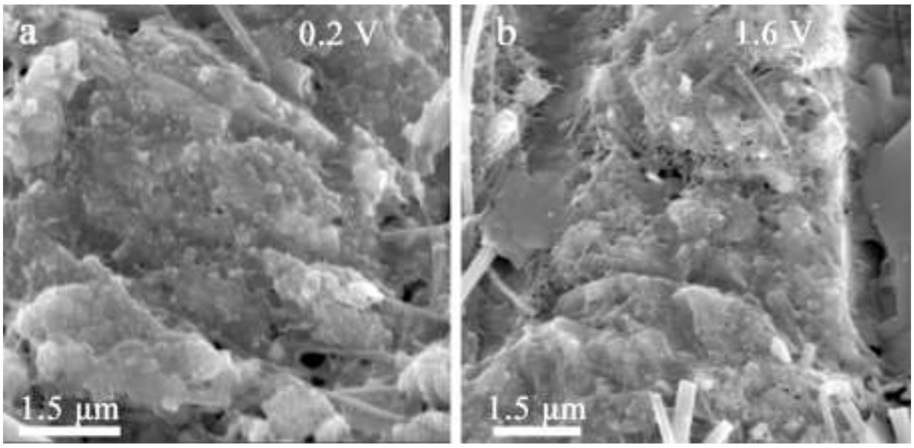

Figure S6 a) SEM of MnVOH at discharged to 0.2 V; b) SEM of MnVOH at charged to 1.6 V.

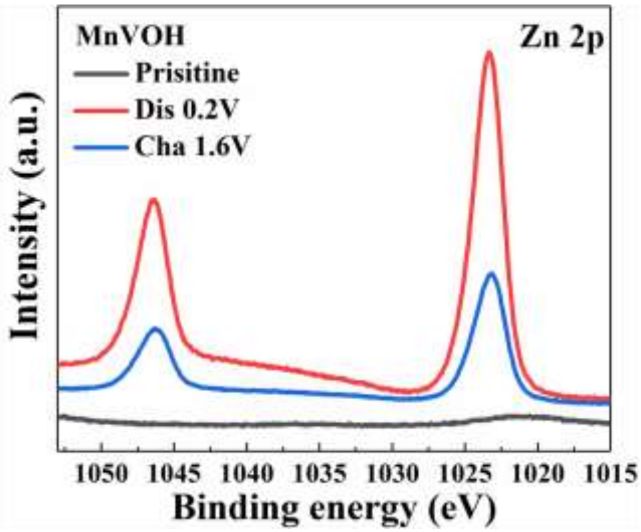

Figure S7 The XPS spectrum of Zn 2p for MnVOH at the discharged to 0.2 V and charged to 1.6 V.

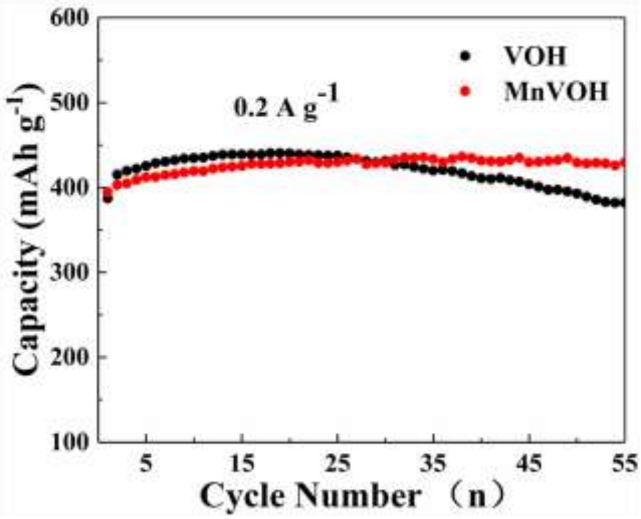

Figure S8 Cycling stability tested at 0.2 A g^{-1} for VOH and MnVOH electrode.

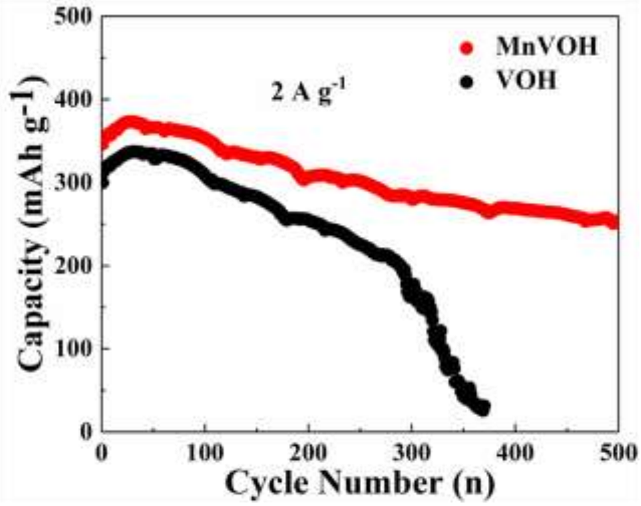

Figure S9 Cycling stability tested at 2 A g^{-1} for VOH and MnVOH electrode.

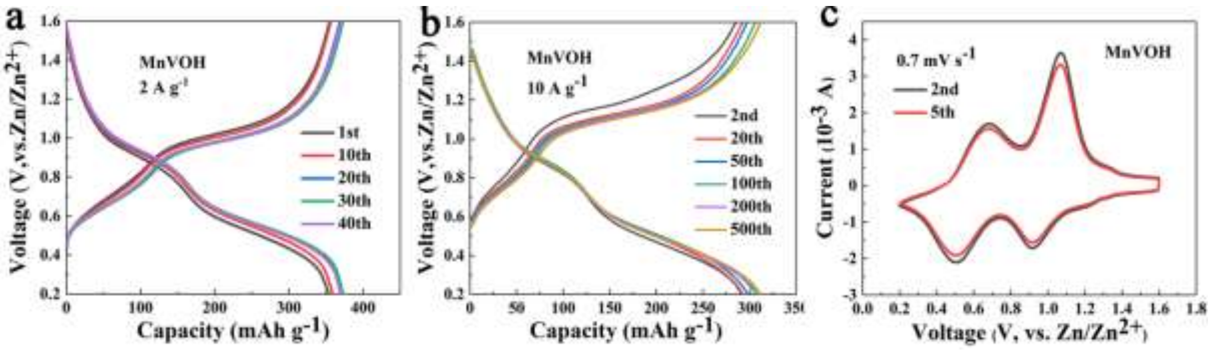

Figure S10 a,b) Different dis/charge curves of MnVOH at 2 A g^{-1} and 10 A g^{-1}. c) CV curves of MnVOH collected at 2nd and 5th, 0.7 mV s^{-1}. The gradual increase in capacity may be caused by the gradual penetration of the electrolyte. The constant GCD and CV curve prove that the reaction mechanism has not changed.

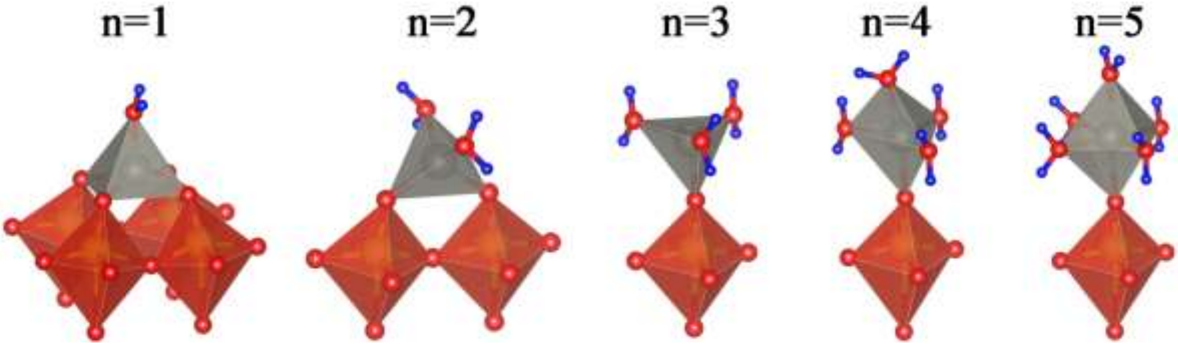

Figure S11 Configurations of $Zn(H_2O)_n^{2+}$ with n=1,2,3,4 and 5.

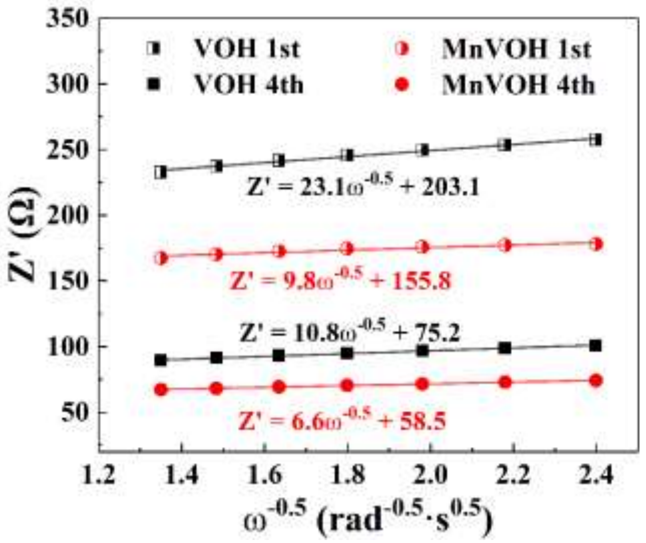

Figure S12 The relationship between the real part of impedance and low frequencies. A smaller slope indicates a higher mobility coefficient.

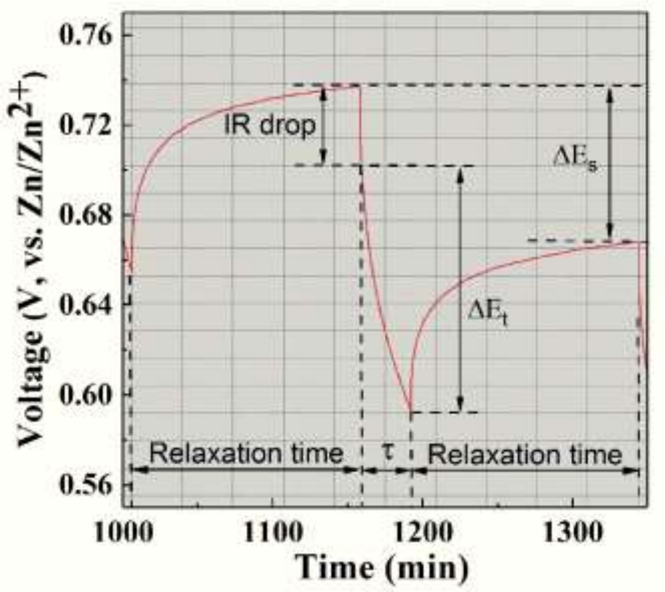

Figure S13 E vs. t curves of KMO electrode for a single GITT during discharge process.

The solid diffusion coefficient was measured by using Galvanostatic Intermittent Titration Technique (GITT) and calculated based on Eq. as follows:[5]

$$D = \frac{4L^2}{\pi\tau}\left(\frac{\Delta E_s}{\Delta E_t}\right)^2$$

Where t is the duration of the current pulse (s), τ is the relaxation time (s), and ΔE_s is the steady state potential change (V) by the current pulse. ΔE_t is the potential change (V) during the constant current pulse after eliminating the iR drop. L is ion diffusion length (cm); for compact electrode, it is equal to thickness of electrode.[6]

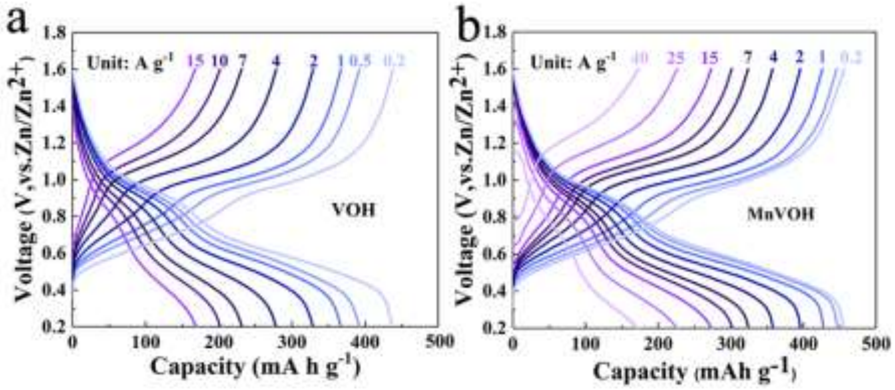

.

Figure S14 Voltage curves at different current densities of VOH (a) and MnVOH (b).

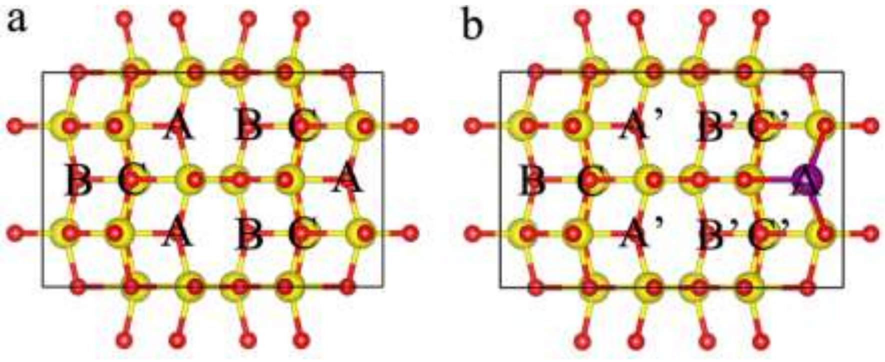

Figure S15 a) Geometric structure of VOH. b) Geometric structure of MnVOH. A, B, C and A', B', and C' represent possible adsorption sites for Zn^{2+} or Mn^{2+}. Since the adsorption energy of Mn^{2+} is greater than that of Zn^{2+} at site A, Mn^{2+} may be preferentially adsorbed to A sites.

.

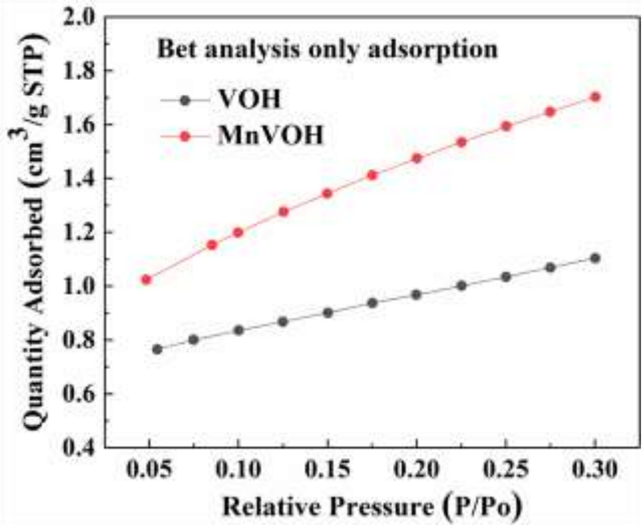

Figure S16 BET analysis only adsorption for VOH and MnVOH. The BET specific surface area of MnVOH is 5.4129 m^2 g^{-1}, the BET specific surface area of VOH is 3.4398 m^2 g^{-1}.

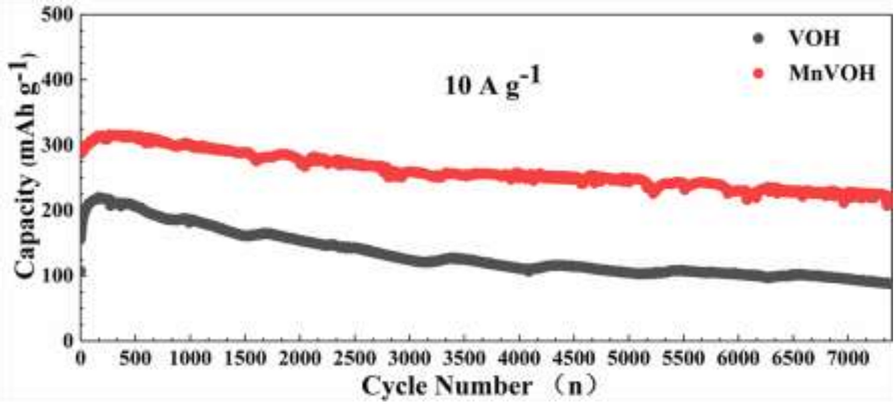

Figure S17 Cycling stability tested at 10 A g^{-1} over 7500 cycles for VOH and MnVOH electrode.

Table S1 The related comparison of Zn^{2+} or Mn^{2+} adsorption on different sites of VOH surface.

Ion Sites	A	B	C
$E_{ads}(Mn^{2+})$/eV	-4.91	-4.69	-4.77
$E_{ads}(Zn^{2+})$/eV	-2.42	-2.86	-2.35

Table S2 The related comparison of Zn^{2+} adsorption on different sites of MnVOH surface.

Ion Sites	B	C	A’	B’	C’
$E_{ads}(Zn^{2+})$/eV	-1.59	-1.92	-2.06	-1.66	-1.66

References

[1] G. Kresse, J. Furthmüller, Phys. Rev. B 1996, 54, 11169.

[2] P.E. Blöchl, Phys. Rev. B 1994, 50, 17953.

[3] H.J. Monkhorst, J.D. Pack, Phys. Rev. B 1976, 13, 5188.

[4] G. Henkelman, B.P. Uberuaga, H. Jonsson, J. Chem. Phys. 2000, 113, 9901-9904.

[5] D. T. Ngo, H. T. T. Le, C. Kim, J.-Y. Lee, J. G. Fisher, I.-D. Kim, C.-J. Park, Energy & Environmental Science 2015, 8, 3577.

[6] G. Fang, C. Zhu, M. Chen, J. Zhou, B. Tang, X. Cao, X. Zheng, A. Pan, S. Liang, Advanced Functional Materials 2019, 29, 1808375.
